# Cloning and activity analysis of the promoter of nucleotide exchange factor gene *ZjFes1* from the seagrasses *Zostera japonica*

**DOI:** 10.1038/s41598-020-74381-6

**Published:** 2020-10-14

**Authors:** Siting Chen, Guanglong Qiu

**Affiliations:** Guangxi Key Lab of Mangrove Conservation and Utilization, Guangxi Mangrove Research Center, Guangxi Academy of Sciences, Beihai, 536007 Guangxi China

**Keywords:** Transcriptional regulatory elements, Molecular ecology

## Abstract

After HSP70 binds to the J domain of the substrate and co-chaperone protein, ATP is hydrolyzed to ADP, and the nucleotide exchange factors (NEFs) promote the release of ADP. Under physiological conditions, the nucleotide exchange step is the rate-limiting step, which is accelerated by NEFs. In this study, the promoter of nucleotide exchange factor *ZjFes1* was cloned, and its expression in tissues and under heat stress was studied to understand the regulatory mechanism of *ZjFes1* and provide the molecular basis to study heat tolerance mechanism of seagrass. It was found that the promoter has common cis-acting elements in promoter and enhancer regions CAAT-box, as well as light response elements AE-box, Box 4 and TCCC-motif, a cis-acting regulatory element essential for the anaerobic induction of ARE, hormone response elements CGTCA-motif and TGACG-motif (MeJA response element), GARE-motif (gibberellin response element), TGA-element (auxin response element), a cis-acting regulatory element related to meristem expression CAT-box, and a cis-acting element involved in defense and stress responsiveness of TC-rich repeats. Two-week-old seedlings exhibited weak GUS activities in their cotyledons. In addition, the *AtFes1A* promoter was constitutively active in the anthers. After exposure to 38 °C for 2 h, the root tips of two-week-old seedlings were stained a strong blue. Heat-inducible activities of GUS were also observed in the cotyledons, roots, leaves, anthers, sepals and siliques.

## Introduction

As is well known, molecular chaperones can help proteins fold in cells^1^. NEFs are critical to the functional cycle of HSP70. Well-studied NEFs include GrpE in *E. coli*^2^, Bag-1^3^ and HspBP-1^4,5^ in animals, and Fes1p in yeast^6^. The yeast Fes1 gene encodes a conserved NEF that acts on cytoplasmic Hsp70s. Mammalian HspBP1 is homologous to Fes1p. HspBP1 promotes the nucleotide dissociation of mammalian Hsc70^4^. HspBP-1 was initially identified as a binding factor to human Hsp70 and was subsequently identified as a functional NEF of cytoplasmic Hsp70, which inhibits the CHIP ubiquitin ligase that directs Hsp70 to the 26S proteasome^7^. When HspBP1 bound to Hsc70, the ubiquitin ligase activity of CHIP decreased^7^. A protein quality control system protects cells from the accumulation of misfolded proteins by promoting selective degradation of misfolded proteins. Hsp70 combines misfolded proteins to facilitate their refolding. If the protein cannot be folded, Hsp70 interacts with ubiquitination enzymes to promote the degradation of misfolded proteins. Hsp70 NEF Fes1 is essential for the ubiquitination of cytoplasmic misfolded proteins, and Fes1 directs Hsp70 substrates to the degradation machinery^8^. Fes1 selectively binds proteins bound to Hsp70 to facilitate their release from Hsp70^8^. In the absence of Fes1, misfolded proteins cannot be polyubiquitinated^8^. As a result, they aggregate and induce a strong heat shock response^8^. Cells maintain protein homeostasis by selectively identifying and degrading misfolded proteins. In *Saccharomyces cerevisiae*, Hsp70 NEF Fes1 is essential for the degradation of misfolded proteins using an ubiquitin–proteasome system. The cytoplasmic splicing variant of Hsp70 NEF Fes1 is essential for the degradation of misfolded proteins in yeast^9^. Fes1 transcripts produce two active isoforms through 3′ alternative splicing^9^. The C-terminus of these two isomers differ and are referred to as Fes1L and Fes1S^9^. Fse1L is located in the nucleus and was the first nuclear Hsp70 nucleotide exchange factor identified^9^. In contrast, Fes1S is located in the cytoplasm, which is a necessary condition for maintaining protein stability^9^. In the absence of Fes1S, the heat shock response was induced under conditions that were normally not stressful^9^. In addition, when the temperature increased, the cells showed severe growth defects^9^. Importantly, misfolded proteins cannot be degraded by the ubiquitin–proteasome system^9^. Genes homologous to HspBP-1 have also been found in other eukaryotes, such as Fes1p in yeast. The deletion of *Fes1* moderately damaged the growth of yeast at 37 °C^6^. Three Fes1p homologues were identified in *Arabidopsis thaliana*^10^, and *AtFes1A* plays an important role in heat response^10^. The expression of AtFes1A was induced by high temperature, and AtFes1A prevented the degradation of Hsp70^10^.

Global climate change is one of the major factors that affects seagrass meadows through its effects on sea level, temperature and CO_2_ in the atmosphere, which can change the distribution and productivity of seagrass^11^. Rising sea surface temperatures impose heat stress on seagrass^12,13^, and the changes in sea surface temperatures directly affect the maintenance of C balance and metabolism in seagrass^14^. In addition, temperature is the main factor that controls the growth of seagrass by altering biochemical processes^15^, where high temperatures inhibit the growth of seagrass^16^.

*Zostera japonica* is a species of seagrass endemic to Asia and is primarily distributed in Japan, Korea and China. *Z. japonica* is the most widely distributed seagrass species in subtropical and temperate coastal areas of China. In the subtropical zone, *Z. japonica* often appears near mangroves or coexists with *Halophila ovalis*. In the temperate zone, *Z. japonica* often lives near *Z. marina* and in the intertidal zone where the water level is shallower than that in which *Z. marina* grows. Only *Z. japonica* can be found in both temperate and subtropical zones in China. *Z. japonica* is an intertidal seagrass, and intertidal seagrass species are more susceptible to heat stress at low tide during the summer months than subtidal seagrass species.

Fes1p has three homologous genes in *Arabidopsis thaliana*, which belong to the ARM (armadillo repeat) superfamily^17^. The fes1p homologous genes of *A. thaliana* are designated *AtFes1A* (*AT3G09350*), *AtFes1B* (*AT3G53800*) and *AtFes1C* (*AT5G02150*). Microarray analysis (Genevestigator, https://genevestigator.com/gv/) showed that among the three *AtFes1* genes, *AtFes1A* was the most significantly expressed by heat induction. A knockout of *AtFes1A* severely impaired acquired thermotolerance in seedlings. Furthermore, AtFes1A and Hsp70 interact in vivo and in vitro. However, AtFes1A has no NEF activity in vitro. Surprisingly, an *AtFes1A* knockout resulted in the down-regulation of Hsp70 protein in cytoplasm and increased transcription of *Hsp*s and *Hsf*s.

Although the homologous genes of hspbp-1/fes1p in *A. thaliana* have been identified, little is known about their homologous genes in seagrass. Promoters control gene expression, and it is important to study the promoter of nucleotide exchange factor in *Z. japonica* to understand regulatory mechanism of the gene. We have previously studied the nucleotide exchange factor of *Z. japonica* and preliminarily verified the function of *ZjFes1*^18,19^, but we did not study the promoter of the gene. In this study, the upstream promoter of *ZjFes1* was cloned using a genome walking method; the promoter sequence was analyzed, and the expression vector was constructed to verify the promoter activity. The promoter activity under heat stress was studied by transforming *A. thaliana* and subjecting the transgenic *A. thaliana* to heat treatment. It is helpful to understand the regulatory mode of *ZjFes1* in *Z. japonica*, and this study provides a theoretical basis to study related nucleotide exchange factors in the future.

## Results

### Cloning and analysis of promoters

**Figure 1:**
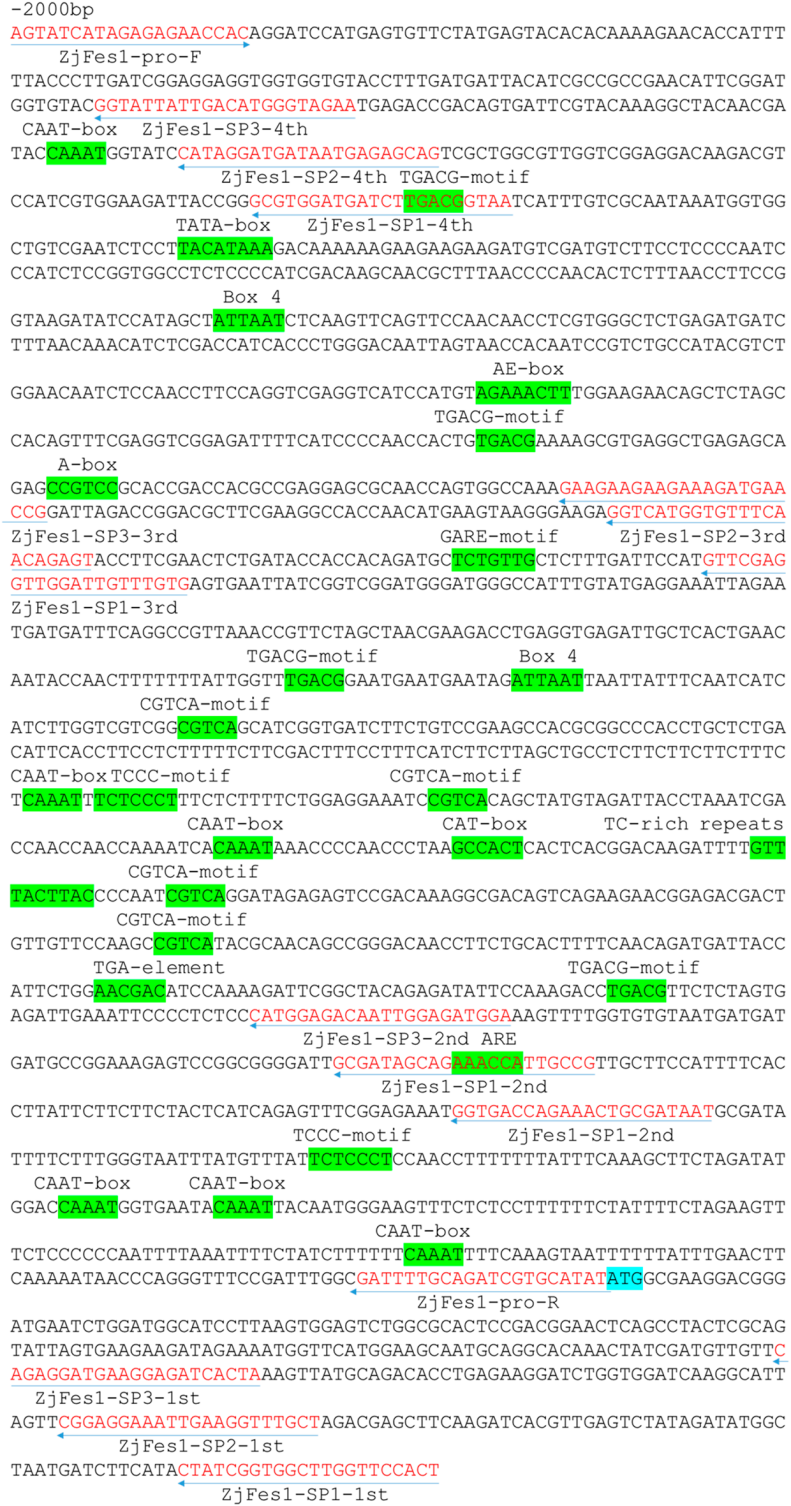
Sequence analysis of the *ZjFes1* promoter. This promoter sequence data of *ZjFes1* was registered in GenBank (No. MN161576). The blue arrow indicates primers used for genome walking and amplification of the full-length promoter sequence. The cis-acting elements are highlighted in green. The initiation codon is highlighted in blue.

According to the primers designed, the 2 kb promoter was obtained by genome walking. Primers were designed at both ends of the promoter, and 2 kb bands were amplified by PCR based on the primers designed. Sequencing results showed that the amplified sequence was identical with the promoter sequence obtained by genome walking, and the sequence could be used in the next experiment. The cloned 2000 bp sequence was predicted online, and the results showed that *ZjFes1* promoter not only contained common cis-acting elements in promoter and enhancer regions CAAT-box but also the light response elements AE-box, Box 4 and TCCC-motif, a cis-acting regulatory element essential for the anaerobic induction ARE, hormone response elements CGTCA-motif and TGACG-motif (MeJA response element), GARE-motif (gibberellin response element), TGA-element (auxin response element), cis-acting regulatory element related to meristem expression CAT-box, and a cis-acting element involved in defense and stress responsiveness TC-rich repeats (Fig. 1).

### Construction of the plant expression vector

**Figure 2:**
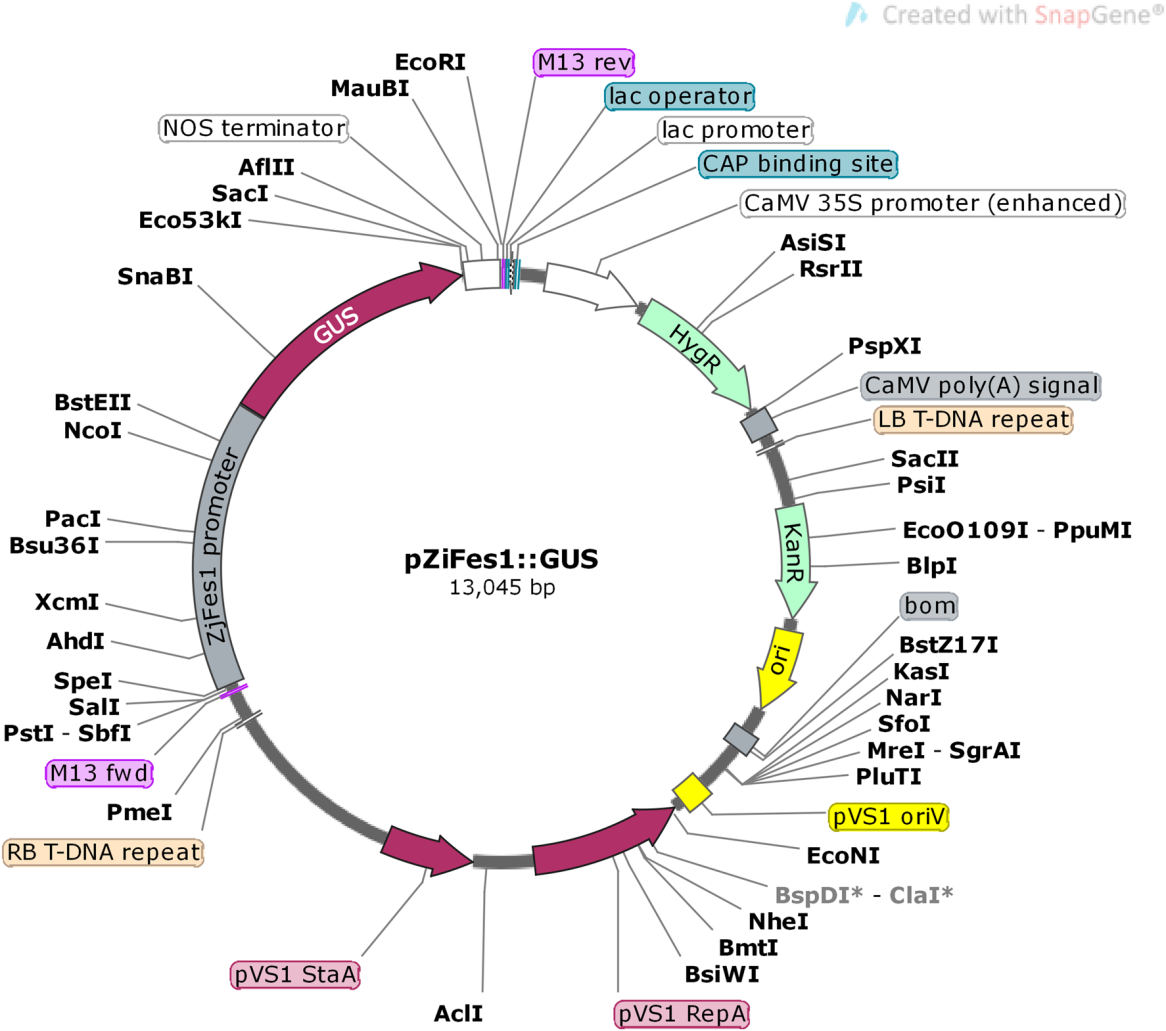
The carrier structure of recombinant plasmid of pZjFes1::GUS.

The promoter fragment obtained (2000 bp) was added with tail A and connected to vector pCXGUS-P/pXcmI-GUS (only GUS, no promoter) to transform *E. coli*. The recombinant plasmid was identified by PCR, which indicated that *ZjFes1* promoter had been inserted into pCXGUS-P/pXcmI-GUS (Fig. 2).

### *Agrobacterium tumefaciens*-mediated transformation of *Arabidopsis thaliana* and GUS staining

**Figure 3:**
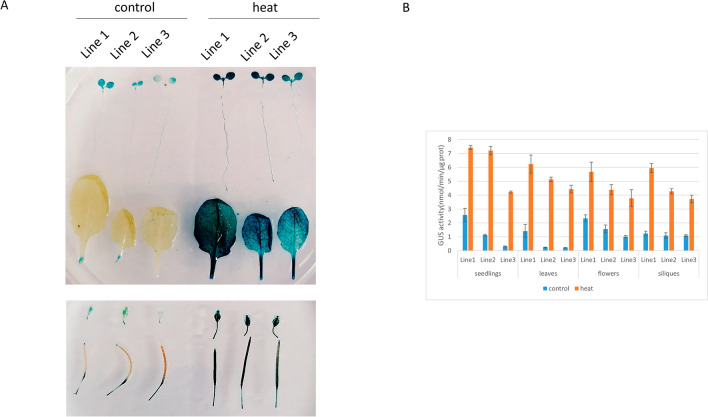
GUS dyeing and activity analysis. (**A**) Illustration of the GUS activities driven by *ZjFes1* promoter in different tissues of transgenic *Arabidopsis*. GUS activities expressed constitutively are presented on the left, and those induced by high temperature are located on the right. (**B**) Enzymatic assay of GUS activity in transgenic *Arabidopsis* seedlings, leaves, flowers and siliques under normal and high temperature.

The recombinant expression vector pZjFes1::GUS was transformed into *A. tumefaciens* GV3101. *A. thaliana* was transformed using an *A. tumefaciens*-mediated floral dip method. The homozygous plants of transgenic *Arabidopsis thaliana* were heat-treated and 2-week-old seedlings, leaves, flowers and siliques were stained with GUS. The expression products of GUS could be detected by *A. tumefaciens* transfected into pZjFes1::GUS in *A. thaliana*, which indicated that the cloned *ZjFes1* promoter could drive the expression of downstream gene *GUS*, i.e., the promoter fragment had driving activity. The homologous gene of *ZjFes1* in *A. thaliana* is *AtFes1A* (At3g09350). AtFes1A was constitutively expressed at low levels at 23 °C and was significantly induced by high temperature^10^. Heat treatment was used to verify the expression of *ZjFes1* promoter under heat stress. Two-week-old seedlings exhibited weak GUS activities in their cotyledons. In addition, the *AtFes1A* promoter was constitutively expressed in anthers. After exposure to 38 °C for 2 h, the root tips of two-week-old seedlings were strongly stained blue. Heat-inducible activities of GUS were also observed in the cotyledons, roots, leaves, anthers, sepals and siliques (Fig. 3). This is similar to the expression of *AtFes1A* promoter in *A. thaliana*^10^. In order to quantitatively detect the activity of *Zjfes1* promoter, GUS activity assay was carried out (Fig. 3B). It can be seen from Fig. 3B that the activity of *Zjfes1* promoter under heat treatment is higher than that under normal temperature, which is consistent with the result in Fig. 3A.

## Discussion

The release of ADP is facilitated by NEFs, and the substrate-binding domain (SBD) of Hsp70 returns to the open conformation and releases the substrate. Although there are some redundant functions and expression patterns, microarray analysis (Genevestigator, https://genevestigator.com/gv/) showed that among the three *AtFes1* genes, *AtFes1A* was the most significantly expressed by heat induction. Recent studies have shown that *AtFes1A* may play a regulatory role in maintaining the stability of Hsp70 and abiotic stress tolerance^10^.

Gene expression in higher plants is primarily regulated by complex factors at the transcriptional level, which are coordinated by many cis-acting elements and trans-acting factors. The promoter is an important cis-acting element in transcriptional regulation and is the center of transcriptional regulation, which determines the temporal and spatial sequence of target gene expression to a certain extent. Therefore, the study of structure and function of promoter is key to understanding molecular mechanism of plant gene expression regulation. *Z. japonica* is an intertidal seagrass, and intertidal seagrass species are more susceptible to heat stress at low tide during the summer months compared with subtidal seagrass species.

In this study, the *ZjFes1* promoter was cloned from genome of *Z. japonica*. A sequence analysis showed that the promoter not only contained common cis-acting elements in promoter and enhancer regions CAAT-box but also light response elements AE-box, Box 4, and TCCC-motif, a cis-acting regulatory element essential for the anaerobic induction ARE, the hormone response elements CGTCA-motif and TGACG-motif (MeJA response element), GARE-motif (gibberellin response element), TGA-element (auxin response element), a cis-acting regulatory element related to meristem expression CAT-box, and a cis-acting element involved in defense and stress responses of TC-rich repeats. The prediction of these cis-acting elements can provide a theoretical basis for the study of this gene regulation pattern. Since the GUS signals driven by the promoter of *ZjFes1* as examined in the transgenic *A. thaliana* plants were induced by heat treatment, a typical heat-responsive element such as HSE could be contained in the promoter region. However, no such HSE element was found in the promoter region. In our previous study, to determine whether *ZjFes1* expression was influenced by heat stress, we measured *ZjFes1* mRNA levels 1 h after heat treatment at 40 °C and compared the measurements to plants maintained at the control temperature of 25 °C. Three independent biological replicates were conducted, and we found that the expression of *ZjFes1* increased significantly (approximately 34.53-fold) at 1 h after treatment^18^. The result of GUS staining was consistent with that of qRT-PCR. The promoter of *Zjfes1* may contain a heat-responsive element that has not yet been identified.

This study determined the activity of *ZjFes1* promoter and its expression in seedlings, leaves, flowers and siliques under heat stress. The results showed that *ZjFes1* might play a role in the heat tolerance of *Z. japonica*.

## Material and methods

### Plant material

*Z. japonica* used in this study was collected from Fangchenggang, Guangxi, China.

### DNA extraction and primer design

Leaves of *Z. japonica* were used as materials to extract genomic DNA from young leaves that had grown well. A MiniBEST Plant Genomic DNA Extraction Kit (TaKaRa, 9768) was used to extract genomic DNA from the leaves of *Z. japonica* following the manufacturer’s instructions. Based on the full-length cDNA sequence of *ZjFes1* obtained by RACE^18^, three identical and high annealing temperature specific primers (SP Primer) were designed, and four specifically designed degenerate primers, AP1, AP2, AP3 and AP4, were used for thermal asymmetric interlaced PCR (TAIL-PCR). Typically, at least one of these degenerate primers can react with specific primers by TAIL-PCR based on the difference of annealing temperature, and the flanking sequence of known sequence can be obtained by three nested PCR reactions. Because the length obtained in one experiment cannot meet the experimental requirements, we continue to acquire the flanking sequence according to the sequence information obtained in the first genome walking. Four genome walkings were conducted. Twelve SP Primers were designed. DNAMAN software was used to combine the four fragments described above into a consensus sequence by combining overlapping fragments. Specific primers were designed to amplify 2 kb sequences according to the results (Table 1), and the experimental results were verified.

**Table 1:** PCR primer sequences.

Primer name	Primer sequence (5′–3′)	Annealing temperature (°C)
ZjFes1-SP1-1st	AGTGGAACCAAGCCACCGATAG	62
ZjFes1-SP2-1st	AGCAAACCTTCAATTTCCTCCG	56
ZjFes1-SP3-1st	TAGTGATCTCCTTCATCCTCT	62
ZjFes1-SP1-2nd	ATTATCGCAGTTTCTGGTCACC	58
ZjFes1-SP2-2nd	CGGCAATGGTTTCTGCTATCGC	62
ZjFes1-SP3-2nd	TCCATCTCCAATTGTCTCCATG	58
ZjFes1-SP1-3rd	CACAAACAATCCAACCTCGAAC	58
ZjFes1-SP2-3rd	ACTCTGTTGAAACACCATGACC	58
ZjFes1-SP3-3rd	CGGTTCATCTTTCTTCTTCTTC	56
ZjFes1-SP1-4th	TTACCGTCAAGATCATCCACGC	60
ZjFes1-SP2-4th	CTGCTCTCATTATCATCCTATG	56
ZjFes1-SP3-4th	TTCTACCCATGTCAATAATACC	55
ZjFes1-pro-F	AGTATCATAGAGAGAACCAC	56
ZjFes1-pro-R	ATATGCACGATCTGCAAAATC	55

### Cloning and construction of the plant expression vector and sequence analysis of promoter

The full-length promoter sequence was amplified using high fidelity polymerase 2 × TransStart FastPfu PCR SuperMix (-dye) (TRANSGEN BIOTECH, AS221-01) using the DNA of *Z. japonica* as a template following the manufacturer’s instructions. The PCR products were detected using 1% gel electrophoresis. The results showed that the size of the bands was the same as that of the target fragments, and the PCR products were recovered using a MiniBEST Agarose Gel DNA Extraction Kit Ver. 4.0 (TaKaRa, 9762). The pCXGUS-P plasmid is a vector designed to detect the activity of plant promoters. The promoter activity is detected by the dyeing intensity of GUS. We used *Xcm*I to digest the empty vector to obtain T vector. After recovery, the product was recombined with T vector, and then the recombinant vector was transformed into *E. coli* DH5α Competent Cells (TaKaRa, 9057) following the manufacturer’s instructions. The positive samples identified by PCR were verified by sequencing at the Guangzhou Sequencing Department of Invitrogen. The sequencing results were compared using DNAMAN software. The plasmid was extracted from the correct bacterial solution and designated pZjFes1::GUS. The sequence analysis of cis-acting elements that could possibly be found in the promoter was performed using the plant-CARE online prediction database (plant cis-acting regulatory element, https://bioinformatics.psb.ugent.be/webtools/plantcare/html/)^20^.

### Agrobacterium-mediated genetic transformation of pZjFes1::GUS into *Arabidopsis thaliana*

The fusion vector pZjFes1::GUS was transformed into *Agrobacterium Rhizobium* strain GV3101 chemically competent cells (Biomed, BC304) using the freeze–thaw method following the manufacturer’s instructions. Transgenic plants of *A. thaliana* were obtained by floral dipping. Plants in nutrient soil were cultured to form a large number of immature flower clusters. The monoclone of *A. tumefaciens* GV3101 was selected and inoculated in liquid LB medium containing kanamycin and rifampicin (50 µg/mL). The monoclone was cultured overnight at 200 rpm and 28 °C. A volume of 2 mL bacterial solution was transferred to a 500 mL flask culture (containing 200 mL liquid LB with 50 µg/mL kanamycin and rifampicin added) and was cultured overnight at 200 rpm and 28 °C. The next day, the OD_600_ of *Agrobacterium* solution was 1.8–2.0. The solution was centrifuged at 5000 rpm for 15 min at 4 °C. The supernatant was discarded, and the precipitate of *A. tumefaciens* was resuspended in 1/2 volume (100 mL) osmotic medium (1/2 Murashige-Skoog, 5% sucrose, 0.5 g/L MES, 10 µg/mL 6-BA, 200 µl/L Silwet L-77, and 150 µM acetyleugenone, pH 5.7), resulting in an OD_600_ of approximately 1.6. The bacterial solution was adsorbed on the transformed plants using the floral dip method (5 min), wrapped with film to keep it fresh, and cultured overnight, followed by the removal of the film. The plants were cultured until the seeds were ripe, and they were harvested. A mixed disinfectant consisting of 70% ethanol and 30% bleaching water was used to soak the seeds for 3 min, suspend them continuously, and wash them three times with anhydrous ethanol. The dried seeds were evenly dispersed on the surface of solid screening medium containing hygromycin (25 µg/mL). After stratification at 4 °C for 2 days, the seeds were germinated in a light incubator and cultured for 2 weeks at 21 °C and 16 h light/8 h darkness. The development of seedlings and length of roots were used to determine whether they were transformants.

### GUS dyeing and activity analysis

The expression of *GUS* reporter gene in *Arabidopsis* tissues was determined using a GUS staining kit (Solarbio, G3060) following the manufacturer’s instructions. The seedlings, leaves, flowers and siliques to be dyed were immersed in GUS dye solution and incubated overnight at 37 °C. The chlorophyll was removed with 75% ethanol until the background color disappeared completely. The results were documented by photography using a Canon 60d camera.

The material needed to determine Gus enzyme activity was frozen rapidly with liquid nitrogen, and then ground into powder by ball mill. The extraction buffer solution (50 mM NaH_2_PO4 (pH 7.0), 10 mM EDTA, 0.1% Triton X-100, 0.1 (w / v) sodium dodecyl sulfonate, 10 mM β-mercaptoethanol) were added to extract protein. After centrifugation at 4 °C, 12,000 r/min for 10 min, the supernatant was taken as protein extract. The protein concentration was determined by Bradford method. 4-MUG, the substrate of GUS reaction, was added and reacted at 37 °C for 30 min. Fluorescence measurement was carried out under the condition of 365 nm excitation light and 455 nm emission light. Three independent biological repeats were conducted. Finally, the GUS enzyme activity value was calculated according to the relative change of product in unit time.

## Data Availability

All data generated or analyzed during this study are included in this published article. The promoter sequence data of *ZjFes1* was registered in the GenBank (No. MN161576).
